# Mapping dynamic molecular changes in hippocampal subregions after traumatic brain injury through spatial proteomics

**DOI:** 10.1186/s12014-024-09485-6

**Published:** 2024-05-12

**Authors:** Sudipa Maity, Yuanyu Huang, Mitchell D. Kilgore, Abbigail N. Thurmon, Lee O. Vaasjo, Maria J. Galazo, Xiaojiang Xu, Jing Cao, Xiaoying Wang, Bo Ning, Ning Liu, Jia Fan

**Affiliations:** 1 Center for Cellular and Molecular Diagnostics, Tulane University School of Medicine, New Orleans, LA USA; 2 Department of Biochemistry and Molecular Biology, Tulane University School of Medicine, New Orleans, LA USA; 3 Clinical Neuroscience Research Center, Department of Neurosurgery and Neurology, Tulane University School of Medicine, New Orleans, LA USA; 4 Department of Cell and Molecular Biology, Tulane University, New Orleans, LA USA; 5 Tulane Brain Institute, New Orleans, LA USA; 6 Department of Pathology and Laboratory Medicine, Tulane University School of Medicine, New Orleans, LA USA; 7 Department of Pathology, University of Texas Southwestern Medical Center, Dallas, TX USA; 8 Tulane University Translational Sciences Institute, New Orleans, LA USA

**Keywords:** Liquid chromatography–mass spectrometry, Laser microdissection (LMD), Traumatic brain injury, Spatial proteomics, Hippocampus, Acute and sub-acute phase

## Abstract

**Background:**

Traumatic brain injury (TBI) often results in diverse molecular responses, challenging traditional proteomic studies that measure average changes at tissue levels and fail to capture the complexity and heterogeneity of the affected tissues. Spatial proteomics offers a solution by providing insights into sub-region-specific alterations within tissues. This study focuses on the hippocampal sub-regions, analyzing proteomic expression profiles in mice at the acute (1 day) and subacute (7 days) phases of post-TBI to understand subregion-specific vulnerabilities and long-term consequences.

**Methods:**

Three mice brains were collected from each group, including Sham, 1-day post-TBI and 7-day post-TBI. Hippocampal subregions were extracted using Laser Microdissection (LMD) and subsequently analyzed by label-free quantitative proteomics.

**Results:**

The spatial analysis reveals region-specific protein abundance changes, highlighting the elevation of FN1, LGALS3BP, HP, and MUG-1 in the stratum moleculare (SM), suggesting potential immune cell enrichment post-TBI. Notably, established markers of chronic traumatic encephalopathy, IGHM and B2M, exhibit specific upregulation in the dentate gyrus bottom (DG2) independent of direct mechanical injury. Metabolic pathway analysis identifies disturbances in glucose and lipid metabolism, coupled with activated cholesterol synthesis pathways enriched in SM at 7-Day post-TBI and subsequently in deeper DG1 and DG2 suggesting a role in neurogenesis and the onset of recovery. Coordinated activation of neuroglia and microtubule dynamics in DG2 suggest recovery mechanisms in less affected regions. Cluster analysis revealed spatial variations post-TBI, indicative of dysregulated neuronal plasticity and neurogenesis and further predisposition to neurological disorders. TBI-induced protein upregulation (MUG-1, PZP, GFAP, TJP, STAT-1, and CD44) across hippocampal sub-regions indicates shared molecular responses and links to neurological disorders. Spatial variations were demonstrated by proteins dysregulated in both or either of the time-points exclusively in each subregion (ELAVL2, CLIC1 in PL, CD44 and MUG-1 in SM, and SHOC2, LGALS3 in DG).

**Conclusions:**

Utilizing advanced spatial proteomics techniques, the study unveils the dynamic molecular responses in distinct hippocampal subregions post-TBI. It uncovers region-specific vulnerabilities and dysregulated neuronal processes, and potential recovery-related pathways that contribute to our understanding of TBI’s neurological consequences and provides valuable insights for biomarker discovery and therapeutic targets.

**Supplementary Information:**

The online version contains supplementary material available at 10.1186/s12014-024-09485-6.

## Introduction

Traumatic Brain Injury (TBI), a significant global health challenge, is a leading cause of death and disability worldwide, affecting an estimated 60 million individuals globally each year [1]. Despite extensive research spanning decades and numerous therapeutic strategies focusing on neuroprotection, neurovascular regeneration, and neuro-restoration, discovering a truly effective therapy for TBI remains a formidable challenge.

TBI, primarily caused by external forces impacting the head, results in extensive and lasting neuropathological damage through a complex cascade of secondary brain injury, leading to progressive neurological impairment [2]. The intricate nature of TBI is derived from an incomplete understanding of the complex molecular processes within the injured microenvironment. This knowledge gap includes changes at the molecular level in specific micro-regions of the brain after injury. Research has increasingly focused on the molecular changes specific to certain brain regions, particularly in relation to neurogenesis, synaptogenesis, angiogenesis, and axonal remodeling [3, 4]. TBI significantly influences synaptic plasticity and neurogenesis, two of the pivotal processes that govern brain function and recovery [5]. The hippocampus, a key area for memory, is notably impacted by TBI, particularly in maintaining synaptic plasticity and neurogenesis, often leading to impaired memory functions [6, 7]. The impact on the hippocampus, its subregions, and connected neural pathways can selectively influence specific sensory-modalities, consequently affecting certain memory-related behaviors [1]. These behaviors and altered memory plasticity are commonly reported as future clinical manifestations following TBI, such as parkinsonism [8, 9]. For example, immunohistochemistry results showed that VEGF and tau protein, which account for changes in synaptic plasticity were elevated in the dorsal hippocampus in TBI [10]. In addition, changes in expression levels of MAGI3 observed in hippocampal proteomics post TBI are linked to a wide series of molecular and biological system interactors. These changes may be related to short-term neuroplasticity phenomena or long-term neurodegenerative consequences such as parkinsonism [1]. As a site active in neurogenesis, immature new neurons in the hippocampal dentate gyrus (DG) subregion of mice have been shown to be vulnerable to injury, exhibiting significant degeneration as early as 2 days post-injury [11] and are likely to undergo apoptosis [12]. However, the pathophysiological mechanisms contributing to secondary brain injury in moderate/severe TBI remain largely elusive, primarily due to the tissue and cellular heterogeneity in affected brain regions associated with cognitive impairments and subsequent neurological disorders.

Traditional proteomics and immunohistochemistry have significantly advanced our understanding of TBI pathogenesis [13–15]. However, these methods often lack the spatial resolution necessary to dissect the complex changes that occur at the microenvironment level following TBI. To address this limitation, we have employed mass-spectrometry based spatial proteomics to scrutinize the molecular landscape, mapping the alterations in protein expression within distinct hippocampal subregions during the acute (1 day) and subacute (7 days) phases [16] which are particularly susceptible to long-term neurological deficits following TBI [17]. By unveiling spatially distinct protein expression patterns, our approach aims to illuminate TBI's impact on critical processes associated with the development of neurological disorders, particularly within the various hippocampal subregions. This investigation provides valuable insights that may help pave the way for the development of targeted and effective therapeutic interventions for TBI and the subsequent emergence of neurodegenerative disorders.

## Methods

### Animals

All animal experiments were carried out following National Institutes of Health (NIH) guidelines and all protocols were approved by the Institution Animal Care and Use Committee of the Tulane University School of Medicine (Protocol #2000). C57BL/6J male mice were purchased from Jackson Lab (Stock No: 000664). All experiments were conducted with biological replicates (n = 3). Our study included 9 animals including the three animals in Sham group and 6 experimental injury animals (three in 1-Day post-TBI and three in 7-Day post-TBI).

### Controlled cortical impact model

Controlled Cortical Impact (CCI) models were induced in male C57BL/6J mice (10 weeks old, 25–27 g) using a pneumatically controlled cortical impactor device (Precision Systems and Instrumentation LLC, TBI-0310), following a previously described protocol with slight modification [18]. Any mice deviating from precise parameters such as having unusually small or large brain volumes were excluded from the study. Briefly, mice were anesthetized in a chamber containing 2.5% isoflurane (Anaquest, Memphis, TN, USA) in 70% N_2_O and 30% O_2_, fixed in a stereotaxic apparatus with a gas anesthesia mask using 2% isoflurane delivered through a Fluotec 3 vaporizer (Colonial Medical, Amherst, NH, USA). The scalp was opened to expose the skull and a portable trephine drill (Fine Science Tools, Foster City, CA, USA) was used to create a 5-mm craniotomy on the left cerebral hemisphere equidistant between bregma and lambda. Subsequently, a moderate-severe injury was induced in the ipsilateral cortex and hippocampus, accompanied by pericontusional axonal injury using a 3 mm flat-tip impounder (4.6 m/s impact velocity, 0.65 mm impact depth, and 500 ms duration). Following CCI, the incision was closed with interrupted 4–0 silk sutures, and mice were allowed to recover in their cages. The entire surgical procedure lasted approximately 8 min. Sham mice underwent the same surgical steps, including craniotomy, but without cortical impact, followed by suturing. Mice were anesthetized with 3% isoflurane for 1–2 min and transcardially perfused with 50 mL of ice-cold PBS followed by 30 mL of 4% paraformaldehyde (PFA) in PBS. The brains were rapidly removed and immersed in 4% PFA at 4 °C for 24 h.

### Sectioning and slide preparation

Brains were sectioned using a vibrating microtome (Leica VT1000 E, USA). The dorsal brain region including the cerebellum and the brain stem was trimmed to approximately position upright in the specimen holder, leaving about 1 cm of the cut site above the holder and accessible for sectioning. The specimen holder with an immobilized brain was placed in a buffer tray that was filled with PBS to fully submerge the specimen. Sections of 30 µm thickness were made with the following settings: control knob of knife advancement speed set to 5 and control knob of knife vibration frequency set to 10. Cut sections were immediately transferred to PEN slides with PBS supplemented with 1 U/µl RiboLock RNase Inhibitor (Thermo Fisher Scientific, cat # EO0381).

### Hematoxylin and eosin staining

Brain tissue was rehydrated through a gradient of ethanol dilutions (100%, 95%, 70%; 5 min each) and then washed in diH_2_O for 3 min. Hematoxylin and eosin (H&E) staining was performed using a commercial H&E staining kit (Abcam, #ab245880) following the manufacturer’s recommended protocol, with slight modification to account for slice thickness. Briefly, slides were stained with Mayer’s hematoxylin for 5 min, washed in two changes of diH_2_O, and differentiated in bluing solution for 15 s. Slides were again washed in two changes of diH_2_O, partially dehydrated through a gradient of ethanol dilutions (70%, 95%; 30 s each) and counterstained with eosin Y for 30 s. Excess stain was removed with 95% ethanol and slides were further dehydrated with 100% ethanol (3 × 2 min), cleared with xylene (3 × 5 min), and cover-slipped using Permount medium (Fisher Scientific, SP15-100). Whole slide scanning was performed using an Axio Scan.Z1 Slide Scanner (Zeiss, Oberkochen, Germany).

### Nissl staining

Brain tissue was demyelinated through a series of ethanol dilutions (70%, 95%, 100%, 95%, 70%; 5 min each) and washed in diH_2_O for 3 min. Neuronal Nissl substance was stained using a commercial cresyl violet staining kit (Abcam, ab246816) per the manufacturer’s recommended protocol with minor modification. Briefly, slides were stained in cresyl violet staining solution for 7 min before differentiation in 95% ethanol until all visible cytoplasmic staining was removed. Slides were further dehydrated with 100% ethanol (30 dips) and cleared with xylene (3 × 5 min). Cover slipping and whole slide scanning was performed as above.

### Validation of protein expression by immunofluorescence

Free floating sections were first washed in 1 × PBS for 10 min before being transferred to blocking solution (10% donkey serum, 0.3% Triton X-100 in 1 × TBS) for 1 h. The sections were incubated in primary antibody diluted in incubation solution (5% donkey serum, 0.3% Triton X-100 in 1 × TBS) overnight at 4 °C. Sections were then washed three times in 1 × TBS before being transferred to the proper secondary antibody diluted in incubation solution for one hour at room temperature. Then, sections were again washed three times in 1 × TBS before being mounted to slides. Slides were then cover-slipped using VECTASHIELD Antifade Mounting Medium with DAPI (Vector Labs, H-1200-10) and imaged with an Olympus IX-83 Confocal Laser Scanning Microscope (Olympus Life Sciences, Tokyo, Japan). Antibodies and their dilutions were as follows: mouse anti-GFAP (clone GA5, Millipore, MAB360, 1:100); rat anti-CD44 (clone IM7, Invitrogen, 14-0441-82, 1:200); donkey anti-mouse Alexafluor 488 (Invitrogen, A21202, 1:1000); and donkey anti-rat Alexafluor 488 (Invitrogen, A21208, 1:1000).

### Laser microdissection

Sub-regions of hippocampus were harvested from dry sections of mice brains on PEN slides using the laser microdissection (Leica LMD7, USA) system with Leica Laser Microdissection (LMD)_V 8.3.1 software. The circumferences of regions to be harvested were drawn using the freehand tool. Cutting was done using the ‘Draw and Cut’ function with the following settings: 10× objective, 10% cut speed, 54% cut energy, 90% cut focus, aperture of 3 and specimen balance of 42. The selected tissue regions were catapulted into the cap of microfuge tubes (Non-stick RNase-Free 0.5 ml, Invitrogen, Texas, USA) placed in the tube collector at a working height of—15,356. RNAse-free 0.5 ml collection tubes. Images were taken with DMC45000 cameras, for brightfield images.

### Sample preparation for proteomics

The preparation of microdissected tissue samples was optimized from a previously reported method [19]. Briefly, tissues were mixed with 10 µl lysis buffer (50% Trifluroethanol (TFE), 25 mM ammonium bicarbonate and 5 mM Dithiothreitol) and then reduced at 91 °C for 10 min in a Thermomixer (Eppendorf) and 5 min sonication. This step is followed by alkylation with 25 mM (final concentration) iodoacetamide by incubating for 20 min in the dark. The mixture was added with 50 µl of 25 mM ammonium bicarbonate and digested by trypsin at an enzyme to protein mass ratio of 1:50 overnight at 37 °C. The samples were acidified followed by drying by vacuum centrifugation. The dried pellet was resuspended in 2% acetonitrile and 0.1% formic acid.

### Strong cation exchange (SCX) fractionation

For spectral library generation, the SCX stage tips were prepared, and samples were processed as previously mentioned [19]. Briefly, the tips were activated with 50 µl acetonitrile and the enriched peptides were loaded followed by their elution in 6 buffers: SCX buffer 1: 50 mM ammonium acetate, 20% acetonitrile, 0.5% formic acid; SCX buffer 2: 75 mM ammonium acetate, 20% acetonitrile, 0.5% formic acid; SCX buffer 3: 125 mM ammonium acetate, 20% acetonitrile, 0.5% formic acid; SCX buffer 4: 200 mM ammonium acetate, 20% acetonitrile, 0.5% formic acid; SCX buffer 5: 300 mM ammonium acetate, 20% acetonitrile, 0.5% formic acid; SCX buffer 6: 5% ammonium hydroxide, 80% acetonitrile. All the fractions were dried by vacuum centrifugation and suspended into 0.1% formic acid/2% acetonitrile before injection.

### LC–MS/MS acquisition

Samples were measured using liquid chromatography tandem mass-spectrometry (LC–MS/MS) instrumentation consisting of an Vanquish Neo nanoLC system (Thermo Fisher Scientific) coupled via a nano-electrospray ion source (Thermo Fisher Scientific) to Orbitrap Eclipse™ Tribrid™ MS (Thermo Fisher Scientific). For generation of spectral library, the SCX fractions were loaded onto a 500 bar 300 um × 5 mm C18 trap column and PepMap™ Easy-spray™ C18 (75 um × 500 mm,) analytical column in buffer A (0.1% formic acid in water). The fractions were eluted with a linear gradient from 5 to 25% buffer B (80% acetonitrile, 0.1% formic acid) over 90 min. Following the linear separation, the system was set up to 40% buffer B over 30 min and finally set to 95% buffer B for 20 min, which was followed by re-equilibration to 5% buffer B prior to the subsequent injection. Data were acquired using data-dependent acquisition (DDA) set in 3 field asymmetry ion mobility spectrometry (FAIMS) experiments of − 40, − 55 and − 70. After adjusting each fraction to an estimated 0.5–1.0 μg on column, the fractions were measured at a cycle time of 2 s with 60 s dynamic exclusion. Precursor spectra were collected from 370–1500 m/z at 240,000 resolutions (customized automated gain control (AGC) target of 300, auto max injection time (IT)). The MS/MS were collected on + 2H to + 7H precursors achieving a standard AGC. MS/MS scans were collected using ion trap (with a dynamic IT) with an isolation width of 1.6 m/z and a NCE of 30.

For the DIA acquisitions of the individual sample with a FAIMS voltage at − 50, the peptides were injected using the same chromatography setup and column as mentioned above. The orbitrap was set to acquire 33 scan events of a biological sample pool using 120,000 precursor resolution and 30,000 fragment resolution. The AGC target was set to 3000, with auto IT, the stepped NCE was set to 25, 27, 30, and + 2H was assumed as the default charge state. The DIA was performed with one full MS event followed by 41 MS/MS windows in one cycle resulting in a cycle time of 3 s. Individual samples for proteome profiling acquisitions used single-injection DIA acquisitions was performed using 15 m/z precursor isolation windows in a staggered-window pattern with optimized window placements from 395 to 950 m/z.

### Peptide identification and protein quantification

All DDA data were processed and combined into spectral library by Spectronaut (v17.3) against Mus musculus (Proteome ID UP000000589, 17,196 reviewed entries, accessed in February 2023). Protein, peptide and PSM FDR were set to 0.01. All tolerance parameter was set to dynamic. Trypsin was used as the cleaving enzyme and up to two missed cleavages were allowed. The search engine set cysteine carbamidomethylation as a fixed modification and *N*-acetylation and oxidation of methionine as variable modifications. The data were also searched against a decoy database so that protein identifications were accepted at a false discovery rate (FDR) of 1%. The DIA raw files were analyzed by Spectronaut using the spectral library generated. The XIC extraction window was set to dynamic. Calibration mode was automatic with MZ extraction strategy set to maximum intensity. Precursor cut off was set to 0.01 and single hit was defined by stripped sequence. Quantitation was performed at MS2 level with automatic normalization strategy used by the software.

### Statistical analysis

All statistical analysis was performed in R Studio (version 2022.12.0) unless mentioned otherwise. One-way ANOVA and Fisher’s Least Significant Differences (LSD) test was used to compare groups in each hippocampal sub-regions and generate p values. Proteins were deemed significantly differentially expressed if fold change (FC) ≥ 2 or ≤ 0.5 and p < 0.05. GraphPad Prism (version 10.1.1) was used to generate graphs, heatmaps by using z scores of the protein intensities and dot plots by using number of DEPs and -log_10_ FDR values of each pathway. To investigate protein clusters representing different trends of expression, clustering analysis was performed using Fuzzy c means [20] within R studio, and the degree of clustering ambiguity was set to 1.5.

## Results

### Workflow and quantifying proteomic expression profiles on hippocampus with spatial context

**Fig. 1:**
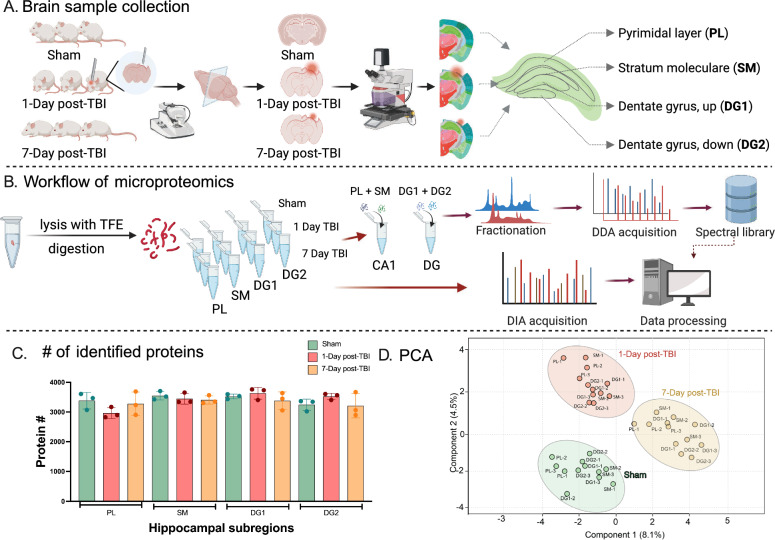
Schematic diagram of the workflow. **A** Brain sample collection. Three mice from each group of Sham, 1-Day and 7-Day post TBI were sectioned in vibratome to obtain the coronal section with fully developed hippocampus. Laser microdissection microscopy was then utilized to cut sub-regions of hippocampus namely pyramidal layer (PL), stratum moleculare (SM), dentate gyrus (top, DG1 and bottom, DG2). Brain section image taken from https://atlas.brain-map.org/. **B** Workflow of micro-proteomics. Trifluroethanol (TFE) was used to lyse tissues followed by reduction, alkylation, and trysin digestion in one tube. PL and SM1 representing cornu ammonis (CA1) of hippocampus were pooled together and DG1 and DG2 were pooled together before subjecting to fractionation by strong cation ion exchange chromatography (SCX). The fractions were analyzed in MS by data-dependent acquisition (DDA) to build a spectral library. The individual sub-regional samples from all groups were analyzed by data-independent acquisition (DIA) and raw files were processed against spectral library. **C** and **D** Data Analysis. **C** Number of identified proteins. Approximately ~ 3500 proteins were identified across each sub-regions and groups and **D** Principal component analysis (PCA) depicts the distinct separation of the three sample types

The study workflow, outlined in Fig. 1, involved the microdissection of hippocampal subregions from three brains each from Sham, 1-Day post-TBI and 7-day post-TBI groups (Fig. 1A). Utilizing laser microdissection, specific hippocampal subregions were precisely collected, including the pyramidal layer (PL) extending to the stratum radiatum, stratum moleculare (SM) of cornu ammonis (CA1), and the DG divided into top (DG1) and bottom (DG2) subregions. Furthermore, CCI achieved reproducible, substantial damage in cerebral cortex post-TBI (Additional file 1: Fig. S1).

Optimization of sample processing was conducted using two methods: sodium dodecyl sulfate (SDS) and TFE initially applied to 3 pooled samples. These samples had a cut size of approximately 50,000 μm^2^ and a 30 μm thick brain section. Tandem mass spectrometry (MS/MS) acquisition methods were optimized using two compensation voltages (CVs) and two collision energies (stepped and fixed) (Additional file 1: Fig. S2A). Among the various methods, the TFE protocol with CV − 50 and stepped collision energy identified approximately 1500 proteins with an injection of approximately 20 ng from a cut of approximately 50,000 μm^2^ and a 30 μm thick brain section (S2A). This method, when applied to individual samples, yielded a comparable number of protein identifications with a ~ 20 ng injection (Additional file 1: Fig. S2A). In terms of peptide identification, the TFE lysate protocol with CV – 50 and stepped collision energy identified over 5000 peptides, which is at least five times higher than the other combinations of CVs and sample preparation strategies (Additional file 1: Fig. S2B). The difference in protein identifications between fixed and stepped collision energy for fragmentation was minimal. An investigation into the feasibility of injecting samples with different CVs was conducted because we employed FAIMS due to its increased selectivity by reducing background noise and allowing better detection of low abundant peptides [21]. Between CV − 35 and CV − 50, the latter identified a greater number of proteins (Additional file 1: Fig. S2C). In contrast, when comparing stepped and fixed collision energy, stepped CE identified more proteins (Additional file 1: Fig. S2D). Consequently, for all samples in the project, the TFE sample protocol with CV − 50 and stepped collision energy was chosen as the preferred method.

The sub-region cuts used for further DIA analysis, were between 200,000–500,000 um^2^ as shown in Additional file 1: Fig. S3A. For three sub-regions (SM, DG1 and DG2), the cut sizes are comparable across all 9 animals within each sub-region. In PL region, two times larger of PL cut were obtained from the Sham group compared to PL cuts from the two TBI groups (Additional file 1: Fig. S3A). Looking into the structure of PL layer, there was a notable shrinkage in TBI groups compared to other subregions (Additional file 1: Fig. S3B). After processing the samples using our optimized protocol, the analysis of all 36 samples was conducted using DIA acquisition. Additionally, six SCX fractions from each of the specified subregions were subjected to DDA to generate a spectral library which successfully identified a total of 4690 proteins (Fig. 1B). Subsequent DIA analysis of the samples identified approximately 3500 proteins from each of the targeted subregions (Fig. 1C, Additional file 2: Table S1). The Principal Component Analysis (PCA) performed on the dataset revealed discernible discrimination among brains from Sham, two TBI groups. The PCA analysis suggests that the proteomic profiles exhibited distinct variations in response to different experimental conditions, thus providing valuable insights into the molecular alterations associated with traumatic brain injury and the subsequent recovery process (Fig. 1D).

### TBI induces differential protein expression pattern and signaling pathways in hippocampal sub-regions

A comprehensive analysis was conducted to map the alterations in protein expression across distinct subregions in the hippocampus-PL, SM, DG1 and DG2 at two distinct time points, 1-Day and 7-Day post-TBI, including Sham group as control.

**Fig. 2:**
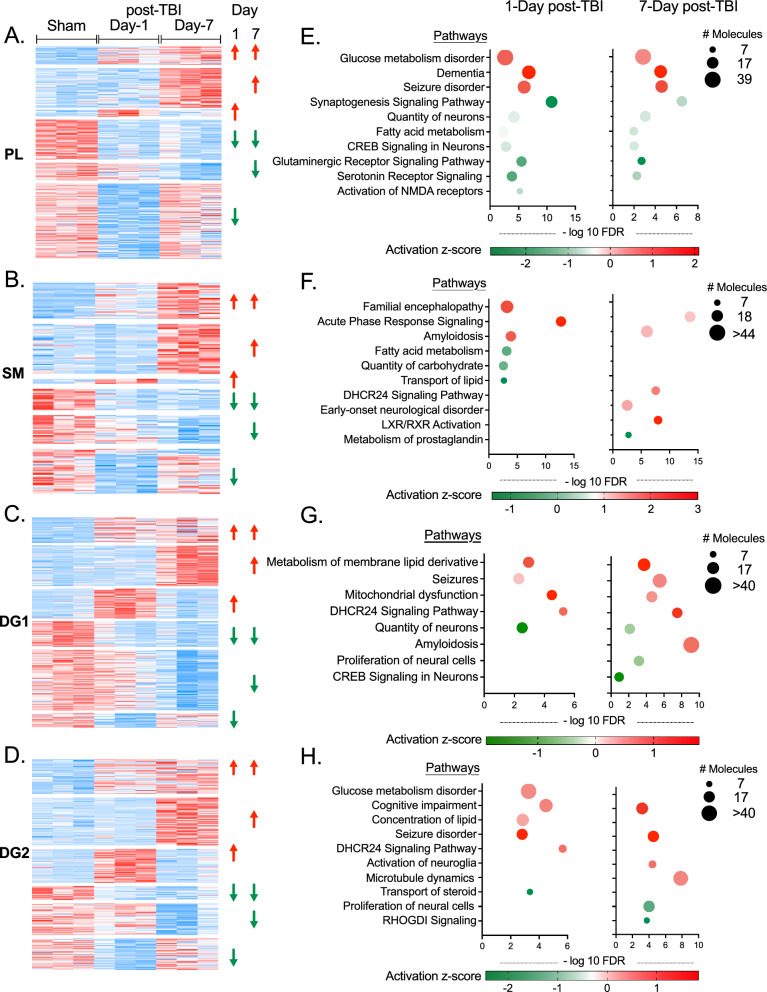
Protein alterations and pathway analysis. **A**–**D** Protein expression pattern. Differentially expressed proteins that showed up-regulation in both 1 day and 7 days post TBI, or either of the time-points, down-regulation in both 1 day and 7 days post TBI, or in either of the time-points in **A** PL; **B** SM; **C** DG1; and **D** DG2. **E**–**F** Pathway enrichment analysis. Ingenuity pathway analysis (IPA) was performed on the differentially expressed proteins and pathways were filtered based on neurological and metabolic pathways. Selection criteria of pathways were the presence of at least 7 proteins with an FDR < 0.05. Pathway enrichment was shown for **E** PL; **F** SM; **G** DG1; and **H** DG2. Criteria of differentially expressed proteins were based on fold change (FC) ≥ 2 or ≤ 0.5 and p < 0.05

Within the PL subregion, 24 proteins exhibited higher expression levels in both 1-Day and 7-Day post-TBI, compared to Sham, while 52 proteins were consistently downregulated at both time points following TBI compared to Sham. A subset of proteins, 10 and 69 respectively, were uniquely upregulated at 1-Day and 7-Day post-TBI. Conversely, exclusive downregulation was noted in 98 and 23 proteins at 1-Day and 7-Day post-TBI, respectively (Fig. 2A). In the SM subregion, 29 proteins increased at time points post-TBI relative to Sham, and 20 proteins decreased in comparison to Sham. In addition, 40 proteins were upregulated, and 24 proteins were demonstrated only at 7-Day post-TBI. There were also changes unique to the Day-1 post TBI, with 4 proteins upregulated and 32 downregulated (Fig. 2B). The analysis of DG1 revealed 40 proteins with higher expression and 41 proteins with lower expression at both 1-Day and 7-Day post-TBI. Furthermore, 64 and 96 proteins exhibited increased and decreased expression exclusively at 7-Day post-TBI, respectively, while 47 and 23 proteins displayed elevated and reduced expression solely at 1-Day post-TBI (Fig. 2C). In DG2, there were 42 proteins consistently increased, and 17 decreased at both time points post-TBI. Additionally, at 7-Day, 58 proteins were uniquely increased, and 38 were decreased, whereas at 1-Day, 41 proteins showed an increase, and 38 a decrease exclusively (Fig. 2D).

Using Ingenuity Pathway Analysis (IPA) to focus on metabolic and neurological pathways, our study identified distinct signaling pathway alterations across these hippocampal subregions post TBI. In PL, synaptogenesis, CREB signaling, glutaminergic and serotonin receptor signaling, along with NMDA receptor signaling, were consistently downregulated at both 1-Day and 7-Day post-TBI (Fig. 2E, Additional file 2: Table S2). This downregulation suggests impairments in synaptic formation and neuronal communication. Upregulated pathways associated with dementia and seizures indicate potential long-term neurological impacts. Disruptions in metabolic pathways were evident, with glucose metabolism disorder upregulated and fatty acid metabolism downregulated, hinting at energy production and utilization issues. In the SM subregions, acute phase response signaling, and amyloidosis were upregulated at both time points. Interestingly, metabolic pathways such as fatty acid metabolism, quantity of carbohydrates and transport of lipids were down-regulated only in 1-Day post-TBI. Notably, 7-days post-TBI saw the dysregulation of pathways such as DHCR24 and LXR/RXR signaling, along with the early onset of neurological disorder and prostaglandin metabolism, indicating secondary and potentially chronic developments (Fig. 2F, Additional file 2: Table S3).

In the two DG subregions, there are shared disruptions across DG1 and DG2, such as DHCR24 signaling, seizures, and reduced neural cells proliferation (Fig. 2G, H, Additional file 2: Tables S4, 5). At 7-Day post TBI, the decline in neural cell proliferation in both DG1 and DG2 suggests potential deficits in long-term potentiation. Additionally, each subregion exhibits unique metabolic pathway dysregulations, for example, DG1 with altered membrane lipid metabolism (Fig. 2G, Additional file 2: Table S4) and DG2 with impaired glucose metabolism, suggesting spatial metabolic variations in subregions (Fig. 2H, Additional file 2: Table S5). Pathways like amyloidosis and CREB signaling are predominant in DG1 (Fig. 2G, Additional file 2: Table S4), whereas DG2 is characterized by changes in microtubule dynamics and steroid transport (Fig. 2H, Additional file 2: Table S5). These variations emphasize spatial dynamics of protein expression and signaling pathway changes post-TBI.

Collectively, these findings contribute to an overall understanding of the temporal dynamics and regional specificity of protein expression alterations and subregion-specific nature of the signaling pathway alterations post-TBI. Other signaling and metabolic pathways marking sub-region differences at different time-points include estrogen receptor signaling, RHO GTPase cycle, MAPK signaling and serotonin receptor signaling (Additional file 2: Tables S2–5).

### Trend analysis of spatial proteomics results based on DG1, DG2, CA1, and PL subregions of mouse brain slices from 1-day post-TBI and 7-day post-TBI models

We applied fuzzy c-means clustering algorithm [22] analysis on proteomic data at different time points post-brain injury to group proteins according to the changes in their expression levels that might be associated with TBI recovery or deterioration. The analysis resulted in 9 different clusters in each sub-region that allowed the construction of dynamic models capturing post-TBI processes, offering valuable insights into the development of various brain diseases (Additional file 1: Fig. S4).

To gain insights into the global change associated with TBI recovery or deterioration, acute and chronic response, we chose the following trends of expression in different sub-regions (clusters were shown in Additional file 1: Fig. S4): consistently up-regulated at both time points (cluster 1, 4 and 5 in PL, cluster 6 and 7 in SM, cluster 2 and 7 in DG1, and cluster 8 and 9 in DG2); up-regulated only at 1 day post-TBI (cluster 8 in PL, cluster 8 in SM, cluster 5 and 6 in DG1, and cluster 3 in DG2) and up-regulated only in 7 days post-TBI (cluster 7 in PL, cluster 9 in SM, cluster 3 in DG1, and cluster 4 and 6 in DG2). Similarly, the down-regulated clusters were grouped as consistently down-regulated in both time points (cluster 6 and 9 in PL, cluster 3, 4 and 5 in SM, cluster 4 and 9 in DG1, and cluster 2 and 7 in DG2); down-regulated only at 1 day post-TBI (cluster 2 in PL, cluster 2 in SM, cluster 1 in DG1, and cluster 3 in DG2) and down-regulated only in 7 days post-TBI (cluster 3 in PL, cluster 1 in SM, cluster 8 in DG1, and cluster 1 in DG2).

**Fig. 3:**
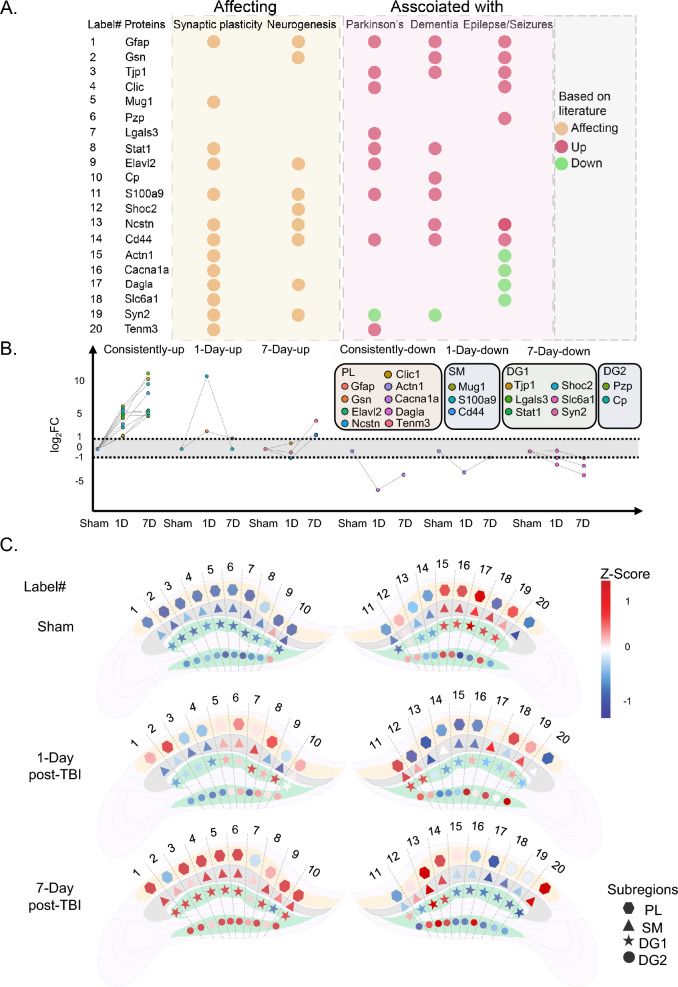
Spatial mapping of proteins in hippocampal subregions. **A** Bubble plot of 20 key proteins. These proteins affect synaptic plasticity or neurogenesis and associated with neurological disorders. **B** Plots of the 20 proteins. The corresponding log_2_FC values were normalized to Sham and plotted in the trend line chart representing their trend of expression in both TBI time points. **C** Cerebral expression maps of the 20 key proteins in 4 subregions at three groups. The hexagonal, triangular, star-shaped and circular point cluster areas represent PL, SM, DG1 and DG2 subregions, respectively. The z-score color scale is from − 1.5 (blue) to 1.5 (red)

Subsequently, proteins were short-listed from these up and down-regulated clusters, based on their functional associations with synaptic plasticity, neurogenesis, and diseases such as Parkinson’s, dementia, and epilepsy from previous literature (Fig. 3A, Table 1) indicated by their trends of expression in the Sham vs. two TBI- time points (Fig. 3B) and spatially mapped based on their expression patterns (Fig. 3C). The roles of these proteins were also supported by studies conducted in mice models or samples collected from humans (e.g., cerebrospinal fluids) at genomics, transcriptomics and/or proteomics level (Table 1). This comprehensive analysis provides valuable insights into differentially expressed proteins in TBI and their potential links to susceptibility to subsequent neurological disorders. For example, proteins consistently up-regulated across all sub-regions, either at both time points (e.g., murinoglobulin-1 (MUG-1) and pregnancy zonal protein (PZP)) or progressively at 7 days post-TBI [e.g., glial fibrillary acidic protein (GFAP), tight junction protein ZO-1 (TJP-1), signal transducer and activator of transcription 1 (STAT-1), CD44 antigen (CD44), and ceruloplasmin (CP)] (Fig. 3C), hint at shared molecular mechanisms in response to TBI-induced stress. These proteins are also found associated with neurological disorders and their overexpression in a maximum time-point of seven days could potentially reflect the predisposition of TBI brain to the neurological disorders (Fig. 3A). To further confirm our results, GFAP and CD44 were selected for validation in tissue sections using immunofluorescence, which showed a consistent trend with proteomics results. These proteins were chosen because blood GFAP is an FDA authorized marker in individuals with suspected TBI [23] and both the proteins have distinguished roles in synaptic plasticity and neurogenesis, with reported associations in Parkinson’s and dementia [24–29]. CD44 was found to be upregulated in 7 days post-TBI compared to 1 day post-TBI and Sham in all sub-regions except SM where it showed upregulation in both the TBI time points as compared to Sham. GFAP consistently showed significant upregulation in 7 days post-TBI compared to Sham and 1 day post-TBI as shown by immunofluorescence and proteomics results (Additional file 1: Fig. S5).

**Table 1:** Functional association of differentially expressed proteins with neurological consequences

Proteins^a^	Plasticity		Neurogenesis		Parkinson’s		Dementia		Epilepsy/seizures	
	Human	Mice	Human	Mice	Human	Mice	Human	Mice	Human	Mice
GFAP	P [30, 31]		P [32]		P [33]		P [26]		P [27]	
GSN				G [34]	P [35]		P [36]			P [37]
TJP1				G [38]				G [39]		P [40]
CLIC						T [41]			P [42]	
MUG1		P [43]								
PZP									P [44]	
LGALS3					P [45]					
STAT1		G [46]				G [47]		G [48]		
ELAVL2		T [49]		T [50]	G [51]					
CP							P [52]			
S100A9		P [53]		P [54]	P [55]		P [56]			
SHOC2				G [57]						
NCSTN		G [58]		G [59]			P [60]			P [61]
CD44		P [24]		G [25]	T [62]		P [28]		G [29]	
ACTN1		P [63]							T [64]	
CACNA1A		G [65]							T [66]	
DAGLA		T [67]		T [67]					T [68]	
SLC6CA1		G [69]							G [70]	
SYN2		G [71]		G [72]	P [73]		G [74]			
TENM3		G [75]			G [76]	G [77]				

^a^Twenty proteins were associated functionally with neuronal plasticity, neurogenesis and neurological disorders like parkinson’s, dementia and epilepsy/seizures. Relevant literature describing the functional association of these proteins either in humans or mice by experiments conducted by genomics (G), transcriptomics (T) or proteomics (P) were cited

Spatial variations were also evident among sub-regions with proteins being dysregulated in both or either of the time-points exclusively in each subregion (Additional file 2: Tables S6, 7). Among these proteins, several were found to influence synaptic plasticity or neurogenesis, two key factors of cognitive functioning. For instance, proteins with overexpression in both time points, such as ELAV like protein 2 (ELAVL2)[50] (Fig. 3B, Additional file 2: Table S6), chloride intracellular channel protein 1 (CLIC1) [78] (Additional file 2: Table S6) in PL, CD44 [79] and MUG-1 [43] in SM (Fig. 3B, Additional file 2: Table S6) and leucine-rich repeat protein SHOC-2 (SHOC2) [57], galectin 3 (LGALS3) [80] in DG1 (Fig. 3B, Additional file 2: Table S6), were associated with synaptic plasticity and subsequent neurodegeneration. Such examples were also evident among time-point-specific proteins such as gelsolin (GSN) [81] and protein—S100A9 [53] overexpressed in 1 day and nicastrin (NCSTN) [58] and teneurin-3 TENM3 [75] exclusively overexpressed at 7 days post-TBI in PL (Fig. 3B, Additional file 2: Table S6). A similar pattern was also observed in downregulated proteins such as voltage-dependent P/Q-type calcium channel subunit alpha-1A (CACNA1A) [82] and sodium- and chloride-dependent GABA transporter 1 (SLC6A1) [83], down-regulated in both time points from PL and SM (Fig. 3B, Additional file 2: Table S7), respectively, along with actinin-1 (ACTN1) [84] down-regulated only in 1 day post-TBI from PL, and diacylglycerol lipase-alpha (DAGLA) [85] downregulated in PL exclusively at 7 days post-TBI (Fig. 3B, Additional file 2: Table S7). These discerned spatial variations in protein expression patterns following TBI, notably those impacting synaptic plasticity and neurogenesis, contribute pivotal insights into the inclination of TBI towards subsequent neurological consequences.

## Discussion

In clinical proteomics studies, tissue heterogeneity is often disregarded due to the requirement for large input material amounts [86]. Conversely, spatial proteomics proves advantageous in elucidating insights into tissue sub-region heterogeneity, thereby revealing molecular alterations specific to these sub-regions in various diseases. This study comprehensively analyzes proteomic expression profiles in spatially located subregions of the hippocampus, shedding light on molecular alterations associated with two time points (1 and 7 days) post-TBI. Sub-region protein expression patterns, unveil distinct variations at different post-TBI time points, potentially linked to long-term consequences. Following LCM, we present a sample preparation regime with MS acquisition workflow to detect protein expression differences with spatial sub-region context in mice brain tissue. Our method, which utilizes tissue sections of approximately 0.5 mm^2^ and a thickness of 30 μm, yields over 3500 protein IDs—a substantial improvement in resolution over a previous study on microproteomics landscape in brain injury, which identified around 2000 proteins from sections of 1 mm^2^ and a thickness of 200 μm [87].

The spatial analysis of protein expression patterns in distinct subregions following TBI provides valuable insights into the heterogeneous molecular response within the hippocampus. Noteworthy are the region-specific abundance changes observed in proteins like fibronectin 1 (FN1), LGALS3BP, haptoglobin (HP), and MUG-1, which were exclusively overexpressed in the SM subregion at both TBI day 1 and day 7 post-TBI. Of these proteins, LGALS3BP is a key player in astrocyte proliferation and neurosphere formation [88], and MUG-1 was previously implicated in neutrophil degranulation [89]. As also evidenced by our pathway analysis, acute phase response signaling was also enriched in SM as compared to other sub-regions. These spatially altered protein expression patterns could possibly indicate the immune cell enrichment in SM following TBI.

Conversely, the over-expression of immunoglobulin heavy constant mu (IGHM) and beta-2-microglobulin (B2M), recognized hallmarks of chronic traumatic encephalopathy [90, 91], specifically in DG2, the deepest region of the hippocampus, underscores the impact of TBI without direct mechanical injury. This observation in DG2 may signify a distinct molecular response to TBI, independent of the mechanical injury, further emphasizing the complexity and heterogeneity of the pathological processes associated with TBI. On a broader scale, the observation of several proteins, including GFAP, signal transducer and STAT1, PZP, CP, VTN, and KN, being overexpressed across all subregions following TBI is also potentially indicative of shared molecular mechanisms in response to TBI-induced stress.

The analysis of metabolic pathways in the aftermath of TBI has unveiled notable disturbances in glucose and lipid metabolism. However, a distinctive observation is the activation of cholesterol synthesis pathways, specifically involving DHCR24 and the LXR/RXR activation, in the SM on post-TBI day 7. Remarkably, these pathways persist in all deeper sub-regions at both TBI time points. This potentially suggests that regions experiencing less mechanical injury might initiate the recovery process, given the critical role of cholesterol synthesis in maintaining brain homeostasis [92]. This restorative trend is further substantiated by the observed activation of neuroglia and microtubule dynamics in DG2 on post-TBI day 7. The coordination of these processes in DG2 signifies a potential mechanism for neural repair and reconstruction, reinforcing the hypothesis that regions less affected by mechanical injury may actively engage in recovery-related pathways.

The cluster analysis has led to the identification of 20 proteins that serve as indicators of dysregulated neuronal plasticity and neurogenesis, crucial consequences of TBI, with potential implications for future neurological disorders like Parkinson’s, dementia, and epilepsy. One prominent protein among these is GFAP, consistently upregulated in all sub-regions at both time points post-TBI. The upregulation of GFAP aligns with its established role as a blood marker for TBI [23], providing robust support that our findings are reliable and confident. Moreover, GFAP has been implicated in various neurological disorders, including dementia, Parkinson’s, and epilepsy, further emphasizing its significance in the context of TBI [93, 94]. Spatial variation was observed by proteins like MUG-1, PZP, and ELAVL2 exclusively being expressed in the PL subregion while S100-A9 showed consistent upregulation in DG1 at both time points. Of these, MUG-1, known as an inhibitor of synaptic plasticity [43], potentially implies its role in disrupting crucial neuronal processes post TBI. On the other hand, PZP and ELAVL2 were known as pyramidal neuron-specific markers linked to Parkinson’s [95, 96]. This implies that the molecular changes identified post-TBI may not only indicate general neuronal dysfunction but also suggest involvement of neuro-pathways and markers in specific sub-regions.

Conversely, downregulated proteins such as SLC17A7 and synapsin-2 (SYN-2), crucial for maintaining synaptic plasticity and neurogenesis [97, 98], were identified. Previous associations of these proteins with diseases like Parkinson's, dementia, and epilepsy support their role in TBI-related processes. Notably, SLC17A7, previously found in hippocampal astrocytes [99] associated with TBI, reinforces the relevance of our discovery. In summary, the short-listed proteins provide valuable insights into the molecular signatures associated with dysregulated neuronal plasticity and neurogenesis following TBI. The links established between these proteins and neurological disorders along with their spatial distribution underscore their potential as biomarkers and therapeutic targets, advancing our understanding of the long-term consequences of traumatic brain injury.

The spatial proteome changes found by our study have the potential to advance the discovery of diagnostic and therapeutic approaches for an early-stage intervention in subsequent neurological disorders such as Parkinson’s disease or dementia, which are associated with TBI. Hippocampal neurons are known to be particularly vulnerable to TBI, leading to cognitive dysfunctions including epilepsy, due to neurodegeneration [100] and injuries to the hippocampus have been observed to impair neurogenesis [101]. Our study further provided localization of DEPs in the hippocampal microregions and their associations with injury pathways and subsequent neuroinflammatory responses. Understanding the spatial distribution of these DEPs can provide valuable insights for identifying therapeutic targets, including inflammation modulation and tissue repair promotion, aimed at ameliorating long-term neurological deficits in TBI patients [87].

Though our study is limited by sample size, it is in line with prior TBI research using mouse models [102, 103]. To properly balance the need for statistical significance with the limited throughput of the spatial proteomics method, we carefully decided to use the current sample size. However, this highlights the need for the development of a more streamlined method for spatial sample proteomics analysis. Our research sheds light on molecular alterations post-TBI during the well-recognized acute and sub-acute phases on day 1 and 7 [16]. However, it is important to acknowledge that the consequences of TBI are long-term, extending beyond these time points. Therefore, future investigations employing longitudinal samples over extended durations could provide deeper insights into the lasting effects of TBI. The scope of this study was centered on spatial proteome changes within the hippocampal sub-regions. Consequently, this focus reveals a limitation of our current study in assessing the effects on other brain regions impacted by TBI, such as the frontal and temporal lobes and/or the cerebral cortex. To address this gap, future research work will be necessary to explore the spatial resolution of protein changes throughout the entire brain post-TBI.

In conclusion, in our study, spatial proteomics unveiled nuanced molecular alterations in distinct hippocampal subregions following TBI at two time points of 1 and 7 days. Specific protein expression patterns in sub-regions highlight unique region-specific vulnerabilities, even in the deepest hippocampal sub-regions without undergoing any direct trauma. Utilizing precise LCM capture and MS, our methodology significantly advances protein identification over previous brain injury studies. Spatial analysis highlights region-specific immune cell enrichment in the SM post-TBI. Metabolic pathway analysis, especially the activation of cholesterol synthesis pathways in DG, suggests potential contribution to neurogenesis and recovery. Cluster analysis has identified 20 proteins indicative of dysregulated neuronal plasticity and neurogenesis post-TBI, including GFAP, an FDA-approved TBI blood marker, and region-specific proteins like MUG-1, PZP, and ELAVL2. This comprehensive analysis provides valuable insights into TBI's dynamic molecular landscape, offering potential biomarkers and therapeutic targets for mitigating long-term consequences.

### Supplementary Information

**Additional file 1: Figure S1.** Three brains, sham, 1 and 7 days post-TBI were stained with Nissl and hematoxylin and eosin (H & E) stain to show the morphological damage to the cortex of TBI as compared to sham. **Figure S2.** A. Comparison was showed between different sample processing strategy and MS acquisition methods. Of all the methods, sample processing with trifluroethanol followed by FAIMS CV voltage of -50 and stepped collision energy yielded the maximum number of proteins. B. The same protocol yielded a corresponding peptide number of 5000 with the individual samples peptide number ranging between 6000–8000. C. Venn diagram depicts the improvement in protein yield with CV 50 as compared to CV 35 and D. using stepped and fixed collision energies. **Figure S3.** A. Microdissection by LCM yielded approximately a cut of 40,000 um^2^ in PL, which was reduced to almost half in post TBI samples. On the contrary, area sections stayed consistent in rest of the sub- regions. B. The Pyramidal layer shows shrinkage on 1 and 7 days post-TBI. **Figure S4.** Clustering of stimulation time expression patterns in identified proteins from (A) DG1, (B) DG2, (C) SM1 and (D) PL subregions with 9 kinds of rising or falling patterns using the fuzzy c-means algorithm. Warm and cold colors indicate low and high deviation from the consensus profile, respectively. **Figure S5.** Immunofluorescence was performed by using CD44 and GFAP antibody on mice brain sections belonging to Sham, and 1-day post-TBI and 7-day post-TBI groups. CD44 showed significant upregulation at day 7 for all sub-regions except SM which also showed upregulation in day 1. This observation was also supported by our proteomics results from different sub-regions at different time-points. We observed similar correspondence between the proteomics and the IHC dataset of GFAP which showed prominent expression on day-7 as compared to day-1 and sham. ns – not significant; * p < 0.05.

**Additional file 2: Table S1.** Proteins identified by Spectronaut. **Table S2.** IPA analysis of differentially expressed proteins in PL. **Table S3.** IPA analysis of differentially expressed proteins in SM. **Table S4.** IPA analysis of differentially expressed proteins in DG1. **Table S5.** IPA analysis of differentially expressed proteins in DG2. **Table S6.** Up-regulated proteins spatially identified in each sub-region and either at both or at each time-point. **Table S7.** Down-regulated proteins spatially identified in each sub-region and either at both or at each time-point.

## Data Availability

Raw mass spectrometry data and results are available in PRIDE repository with identifier PXD051093. All data generated and analyzed during this study are included in the additional files.

## Abbreviations

TBI: Traumatic brain injury
PL: Pyrimidal layer
SM: Stratum moleculare
CA1: Cornu ammonis
DG: Dentate gyrus
CCI: Controlled cortical impact
PFA: Paraformaldehyde
H&E: Hematoxylin and eosin
SCX: Strong cation exchange chromatography
TFE: Trifluoroethanol
LC–MS/MS: Liquid chromatography–tandem mass-spectrometry
DDA: Data-dependent acquisition
DIA: Data-independent acquisition
FAIMS: Field asymmetry ion mobility spectrometry
AGC: Automatic gain control
IT: Ion injection time
NCE: Normalized collision energy
PSM: Peptide spectrum match
FDR: False discovery rate
SDS: Sodium dodecyl sulfate
CV: Compensation voltage
PCA: Principal component analysis
IPA: Ingenuity pathway analysis
