# Supplementary material for: Mapping dynamic molecular changes in hippocampal subregions after traumatic brain injury through spatial proteomics
Source: Clin Proteomics. 2024 May 12;21:32. doi: 10.1186/s12014-024-09485-6 (PMC11089002; doi:10.1186/s12014-024-09485-6)
Supplement: Supplementary file 1 — Additional file 1: Figure S1. Three brains, sham, 1 and 7 days post-TBI were stained with Nissl and hematoxylin and eosin (H & E) stain to show the morphological damage to the cortex of TBI as compared to sham. Figure S2. A. Comparison was showed between different sample processing strategy and MS acquisition methods. Of all the methods, sample processing with trifluroethanol followed by FAIMS CV voltage of -50 and stepped collision energy yielded the maximum number of proteins. B. The same protocol yielded a corresponding peptide number of 5000 with the individual samples peptide number ranging between 6000–8000. C. Venn diagram depicts the improvement in protein yield with CV 50 as compared to CV 35 and D. using stepped and fixed collision energies. Figure S3. A. Microdissection by LCM yielded approximately a cut of 40,000 um2 in PL, which was reduced to almost half in post TBI samples. On the contrary, area sections stayed consistent in rest of the sub- regions. B. The Pyramidal layer shows shrinkage on 1 and 7 days post-TBI. Figure S4. Clustering of stimulation time expression patterns in identified proteins from (A) DG1, (B) DG2, (C) SM1 and (D) PL subregions with 9 kinds of rising or falling patterns using the fuzzy c-means algorithm. Warm and cold colors indicate low and high deviation from the consensus profile, respectively. Figure S5. Immunofluorescence was performed by using CD44 and GFAP antibody on mice brain sections belonging to Sham, and 1-day post-TBI and 7-day post-TBI groups. CD44 showed significant upregulation at day 7 for all sub-regions except SM which also showed upregulation in day 1. This observation was also supported by our proteomics results from different sub-regions at different time-points. We observed similar correspondence between the proteomics and the IHC dataset of GFAP which showed prominent expression on day-7 as compared to day-1 and sham. ns – not significant; * p < 0.05. [file 12014_2024_9485_MOESM1_ESM.docx]

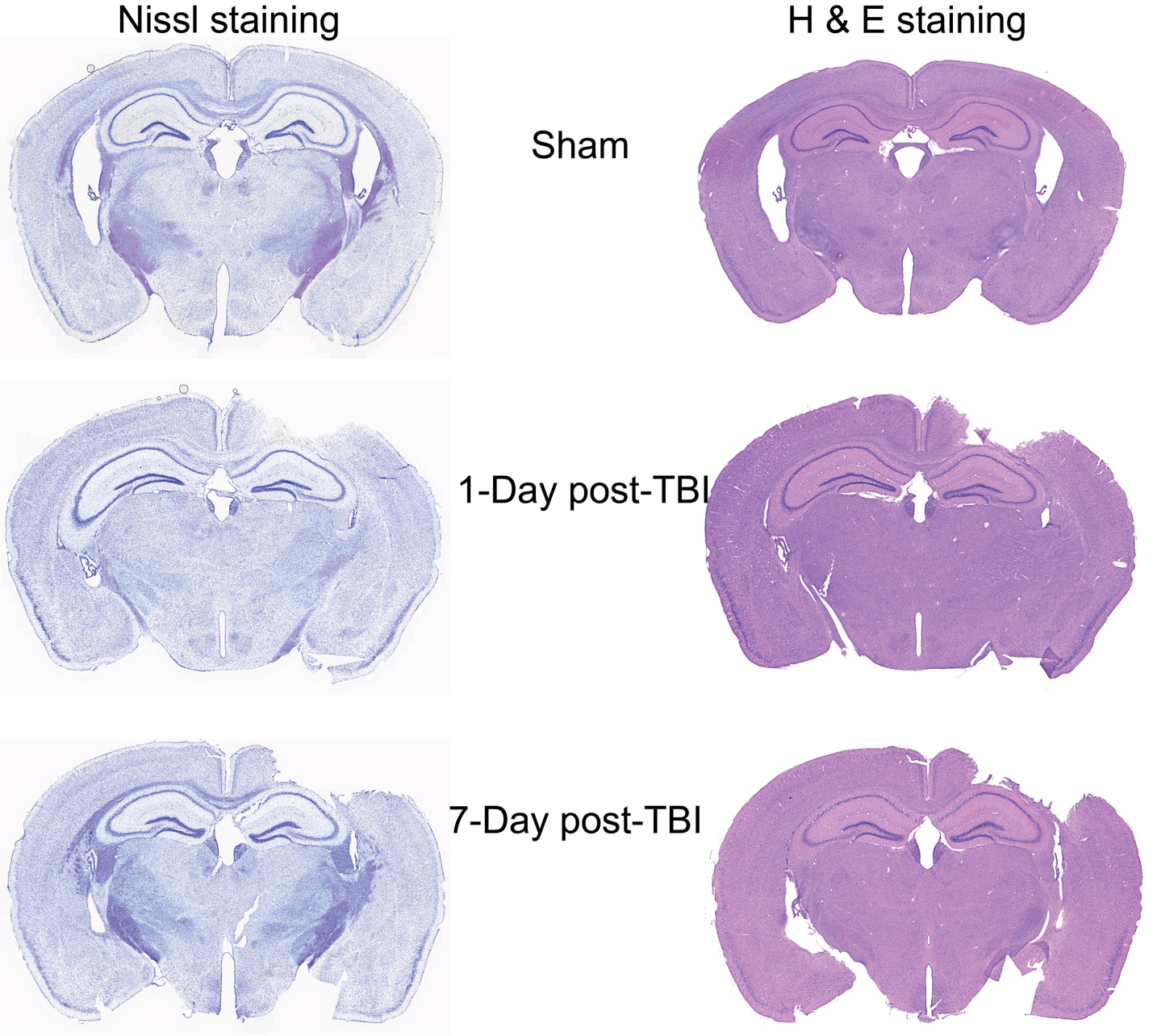


**Figure S1**. Three brains, sham, 1 and 7 days post-TBI were stained with Nissl and hematoxylin and eosin (H & E) stain to show the morphological damage on the cortex of TBI as compared to sham.


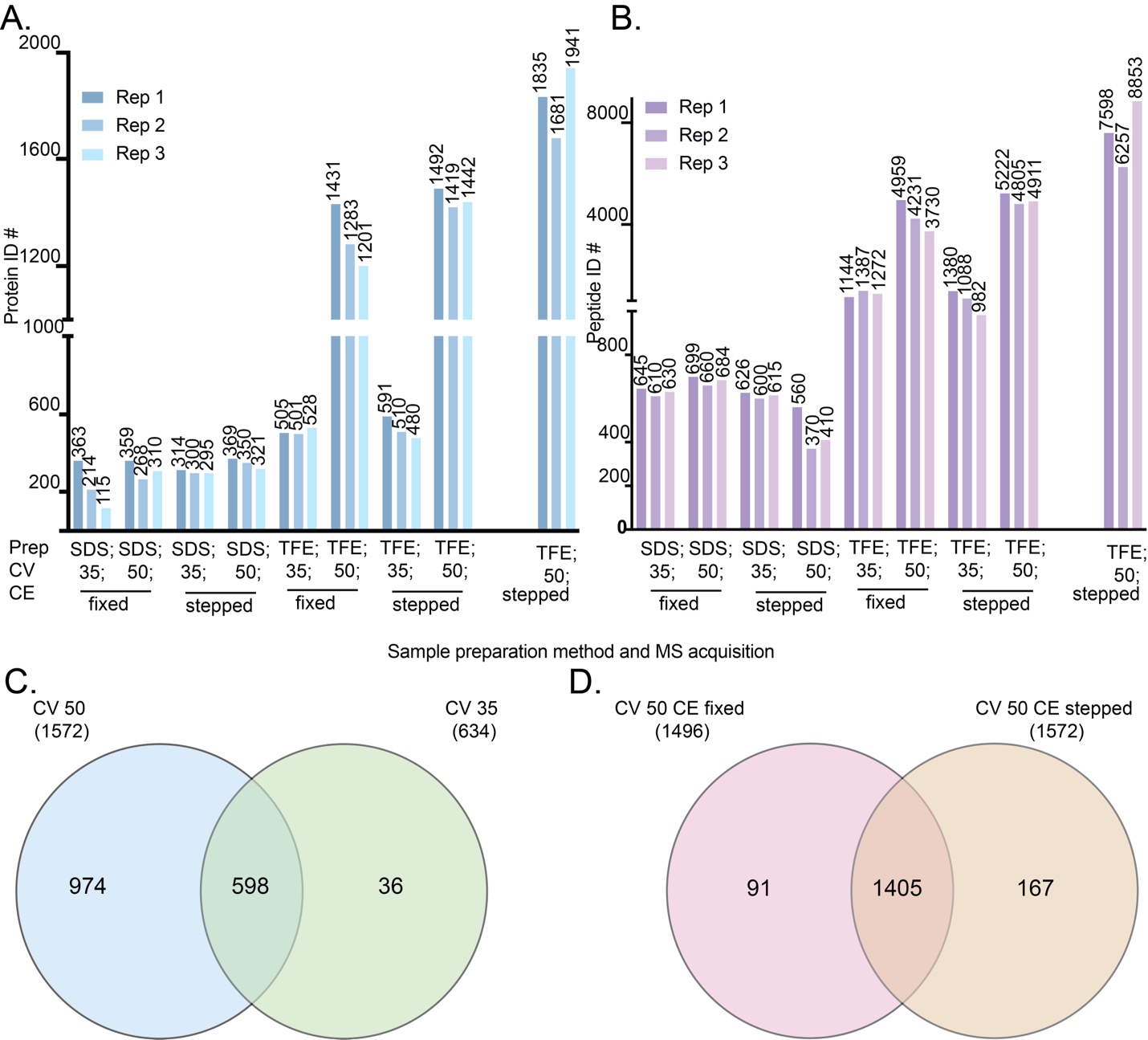


**Figure S2.** A. Comparison showed between different sample processing strategy and MS acquisition methods. Of all the methods, sample processing with trifluroethanol followed by FAIMS CV voltage of -50 and stepped collision energy yielded the maximum number of proteins. B. The same protocol yielded a corresponding peptide number of 5000 with the individual samples peptide number ranging between 6000-8000. C. Venn diagram depicts the improvement in protein yield with CV 50 as compared to CV 35 and D. using stepped and fixed collision energies.


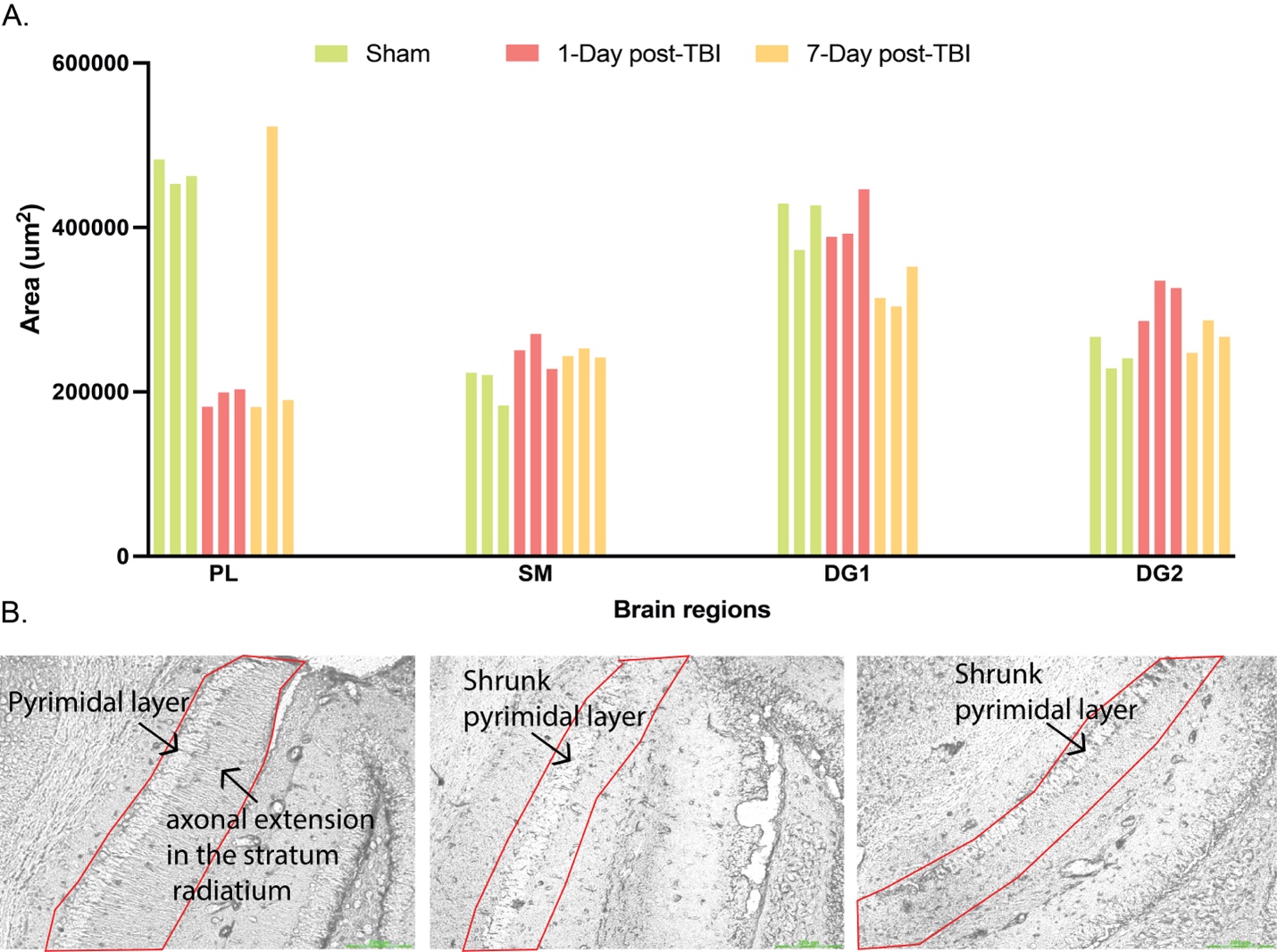


**Figure S3.** A. The microdissection by LCM yielded approximately a cut of 40,000 um^2^ in PL, which was reduced to almost half in post TBI samples. On the contrary, area sections stayed consistent in rest of the sub- regions. B. Pyramidal layer shows shrinkage in 1 and 7 days post-TBI.

**
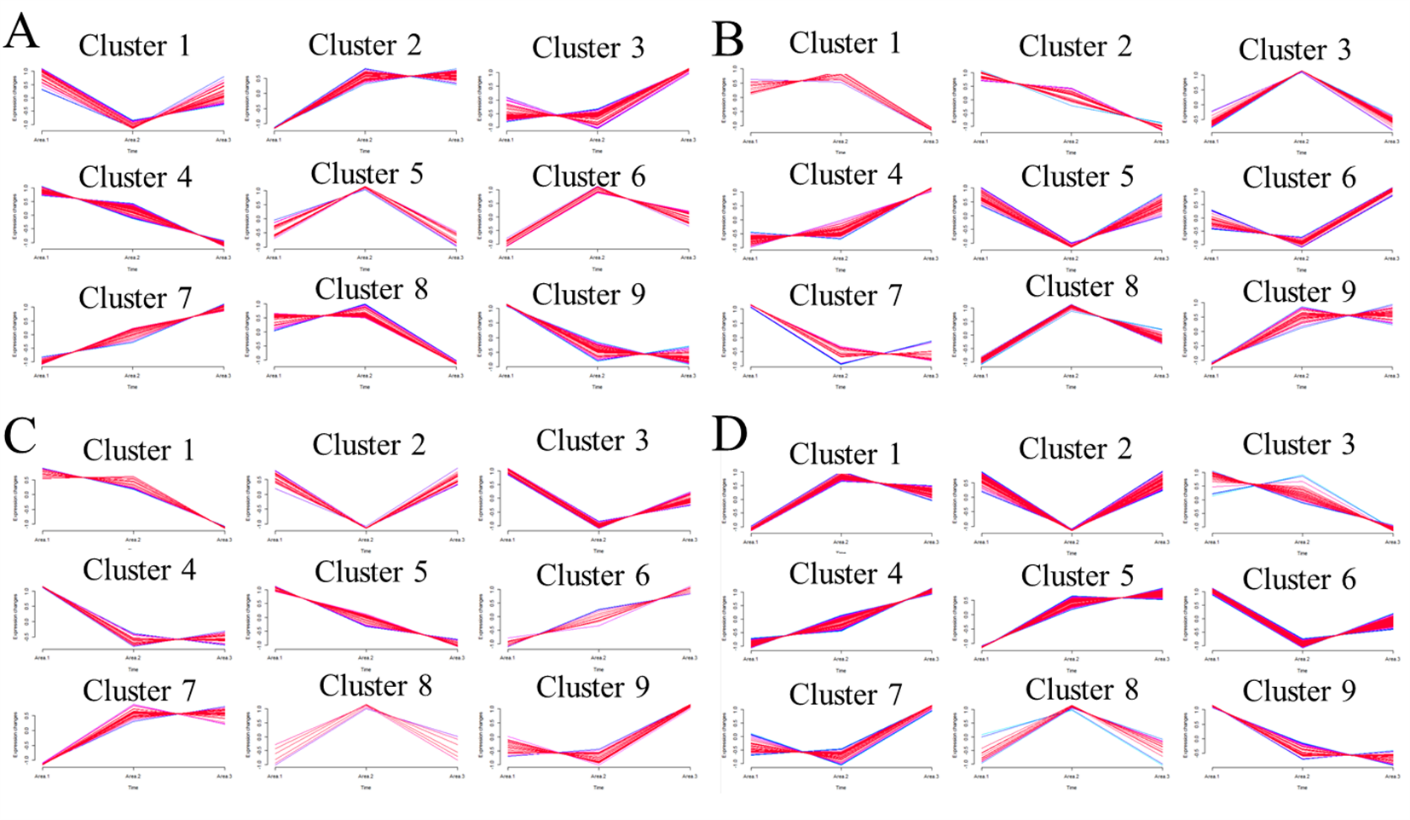
Figure S4.** Clustering of stimulation time expression patterns in identified proteins from (A) DG1, (B) DG2, (C) SM1 and (D) PL subregions with 9 kinds of rising or falling patterns using the fuzzy c-means algorithm. Warm and cold colors indicate low and high deviation from the consensus profile, respectively.


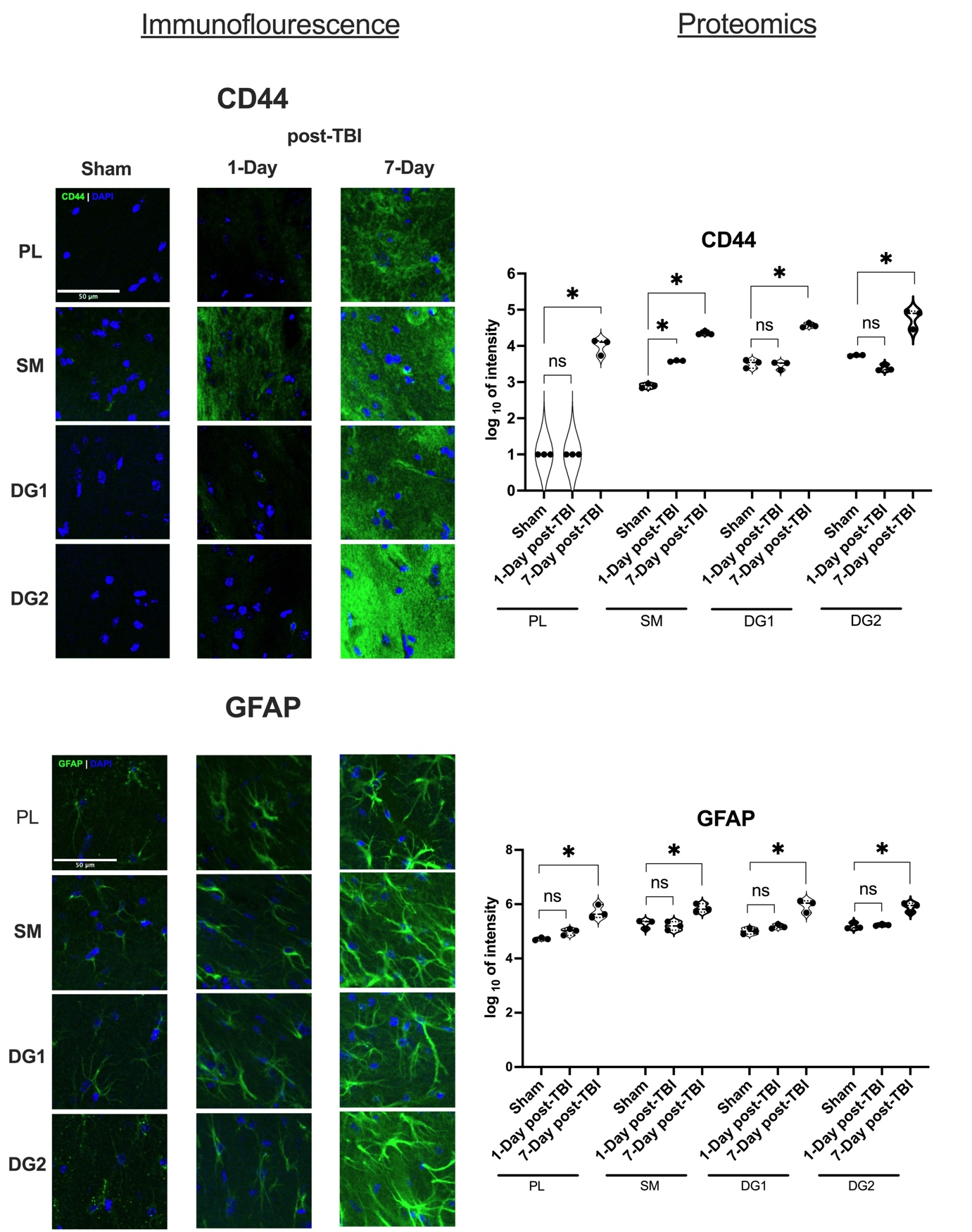


**Figure S5.** Immunofluorescence was performed by using CD44 and GFAP antibody on mice brain sections belonging to Sham, and 1-day post-TBI and 7-day post-TBI groups. CD44 showed significant upregulation at day 7 for all sub-regions except SM which also showed upregulation in day 1. This observation was also supported by our proteomics results from different sub-regions at different time-points. We observed similar correspondence between the proteomics and the IHC dataset of GFAP which showed prominent expression on day-7 as compared to day-1 and sham. ns – not significant; * p<0.05.
